# The role of Effortful Control in Substance Use Disorders

**DOI:** 10.1192/j.eurpsy.2022.173

**Published:** 2022-09-01

**Authors:** E. Santens, G. Dom, E. Dierckx, L. Claes

**Affiliations:** 1 Alexian Psychiatric Hospital, Addiction, Tienen, Belgium; 2 University of Antwerp, Faculty Of Medicine And Health Sciences, Antwerp, Belgium; 3 Vrije Universiteit Brussel (& Alexianen Zorggroep Tienen, psychiatric hospital), Department Of Psychology, Brussel, Belgium; 4 KU Leuven, Faculty Of Psychology And Educational Sciences, Tienen, Belgium

**Keywords:** substance use disorders, Effortful Control, clinical symptoms, Personality disorders

## Abstract

Background and aim: Temperament and personality traits are important factors underlying the vulnerability for both initiation and continuation of addictive behaviors. In SUDs there is a high comorbidity with mood and anxiety disorders and personality disorders. As there is a growing interest in the possible transdiagnostic role of Effortful Control in both SUDs as in their comorbid disorders the current study therefore investigates the influence of reactive (Behavioral Inhibition System and Behavioral Activation System, BIS/BAS) and regulative (Effortful Control, EC) temperament and their interaction in relation to clinical symptomatology and PDs in adult inpatients with SUD. Methods: The EC, BIS/BAS, SCL-90-R and ADP-IV questionnaires were administered to 841 patients (age between 17 y and 71 y) with a SUD admitted at the treatment unit for addiction of a psychiatric hospital. Results: Hierarchical regression analyses showed that both, clinical symptomatology and PDs were related to low levels of effortful control (EC). Furthermore, cluster C PDs were related to high levels of BIS and low levels of EC; whereas cluster B PDs were related to low levels of EC and high levels of BAS for the narcissistic and histrionic PD. An interaction effect (BIS*EC) was found for the Schizotypal PD, all other interactions (BIS*EC and BAS*EC) were not significant. Discussion: We found that low EC is involved in all clinical symptomatology and PDs in a sample of inpatients with SUDs. Therapeutic interventions aiming at strengthening effortful control can possibly result in better treatment outcomes for both the addiction as the comorbid psychopathology

**Disclosure:**

No significant relationships.

